# Effects of a healthy Nordic diet on gene expression changes in peripheral blood mononuclear cells in response to an oral glucose tolerance test in subjects with metabolic syndrome: a SYSDIET sub-study

**DOI:** 10.1186/s12263-016-0521-4

**Published:** 2016-03-17

**Authors:** Lena Leder, Marjukka Kolehmainen, Ingunn Narverud, Ingrid Dahlman, Mari C. W. Myhrstad, Vanessa D. de Mello, Jussi Paananen, Carsten Carlberg, Ursula Schwab, Karl-Heinz Herzig, Lieselotte Cloetens, Matilda Ulmius Storm, Janne Hukkanen, Markku J. Savolainen, Fredrik Rosqvist, Kjeld Hermansen, Lars O. Dragsted, Ingibjörg Gunnarsdottir, Inga Thorsdottir, Ulf Risérus, Björn Åkesson, Magne Thoresen, Peter Arner, Kaisa S. Poutanen, Matti Uusitupa, Kirsten B. Holven, Stine M. Ulven

**Affiliations:** 1 Department of Nutrition, Institute of Basic Medical Sciences, University of Oslo, P.O. Box 1046, Blindern, 0317 Oslo Norway; 2 Institute of Public Health and Clinical Nutrition, University of Eastern Finland, Kuopio, Finland; 3 Department of Medicine (H7), Karolinska Institute, Stockholm, Sweden; 4 Department of Health, Nutrition and Management, Faculty of Health Sciences, Oslo and Akershus University College of Applied Sciences, Oslo, Norway; 5 Institute of Biomedicine, University of Eastern Finland, Kuopio, Finland; 6 Institute of Clinical Medicine, Internal Medicine, Kuopio University Hospital, Kuopio, Finland; 7 Institute of Biomedicine and Biocenter of Oulu, Medical Research Centre Oulu, Oulu, Finland; 8 Department of Gastroenterology and Metabolism, Poznan University of Medical Sciences, Poznan, Poland; 9 Biomedical Nutrition, Pure and Applied Biochemistry, Lund University, Lund, Sweden; 10 Biocenter Oulu, University of Oulu, Oulu, Finland; 11 Institute of Clinical Medicine, Department of Internal Medicine, University of Oulu, Oulu, Finland; 12 Medical Research Center Oulu, Oulu University Hospital and University of Oulu, Oulu, Finland; 13 Department of Public Health and Caring Sciences, Clinical Nutrition and Metabolism, Uppsala University, Uppsala, Sweden; 14 Department of Endocrinology and Internal Medicine, Aarhus University Hospital, Aarhus, Denmark; 15 Department of Nutrition, Exercise and Sport, University of Copenhagen, Copenhagen, Denmark; 16 Unit for Nutrition Research, University of Iceland and Landspitali - The National University Hospital of Iceland, Reykjavik, Iceland; 17 Department of Clinical Nutrition, Skåne University Hospital, Lund, Sweden; 18 Department of Biostatistics, University of Oslo, Oslo, Norway; 19 Research Unit, Kuopio University Hospital, Kuopio, Finland; 20 Norwegian National Advisory Unit on Familial Hypercholesterolemia, Department of Endocrinology, Morbid Obesity and Preventive Medicine, Oslo University Hospital, Oslo, Norway

**Keywords:** mRNA gene expression, Metabolic syndrome, PBMCs, Nordic diet, OGTT

## Abstract

**Background:**

Diet has a great impact on the risk of developing features of metabolic syndrome (MetS), type 2 diabetes mellitus (T2DM), and cardiovascular diseases (CVD). We evaluated whether a long-term healthy Nordic diet (ND) can modify the expression of inflammation and lipid metabolism-related genes in peripheral blood mononuclear cells (PBMCs) during a 2-h oral glucose tolerance test (OGTT) in individuals with MetS.

**Methods:**

A Nordic multicenter randomized dietary study included subjects (*n* = 213) with MetS, randomized to a ND group or a control diet (CD) group applying an isocaloric study protocol. In this sub-study, we included subjects (*n* = 89) from three Nordic centers: Kuopio (*n* = 26), Lund (*n* = 30), and Oulu (*n* = 33) with a maximum weight change of ±4 kg, high-sensitivity C-reactive protein concentration ≤10 mg L^−1^, and baseline body mass index <39 kg m^−2^. PBMCs were isolated, and the mRNA gene expression analysis was measured by quantitative real-time polymerase chain reaction (qPCR). We analyzed the mRNA expression changes of 44 genes before and after a 2hOGTT at the beginning and the end of the intervention.

**Results:**

The healthy ND significantly down-regulated the expression of toll-like receptor 4 (*TLR4*), interleukin 18 (*IL18*), and thrombospondin receptor (*CD36*) mRNA transcripts and significantly up-regulated the expression of peroxisome proliferator-activated receptor delta (*PPARD*) mRNA transcript after the 2hOGTT compared to the CD.

**Conclusions:**

A healthy ND is able to modify the gene expression in PBMCs after a 2hOGTT. However, more studies are needed to clarify the biological and clinical relevance of these findings.

**Electronic supplementary material:**

The online version of this article (doi:10.1186/s12263-016-0521-4) contains supplementary material, which is available to authorized users.

## Background

The metabolic syndrome (MetS) is a cluster of risk factors increasing the risk of type 2 diabetes mellitus (T2DM) and cardiovascular diseases (CVD) (Alberti et al. 2009). Obesity, insulin resistance (IR), and T2DM are associated with chronic low-grade inflammation (Bastard et al. 2006; Wellen and Hotamisligil 2005), which plays a pivotal role in all phases of atherosclerosis (Libby et al. 2009). Diet has a great impact on the risk of MetS, T2DM, and CVD (Alberti et al. 2009; Mozaffarian et al. 2011). Thus, it is crucial to understand the role of diet and dietary compounds on inflammation in the development of these diseases.

A healthy Nordic diet (ND) has been shown to improve lipid profile among hyper-cholesterolemic subjects (Adamsson et al. 2011). The Systems Biology in Controlled Dietary Interventions and Cohort Studies (SYSDIET) study was a multicenter randomized dietary study in individuals with features of MetS. A healthy ND with whole-grain products, berries, fruits and vegetables, rapeseed oil, three fish meals per week, and low-fat dairy products was compared to an average Nordic diet served as control diet (CD) (Uusitupa et al. 2013). In the SYSDIET study, we showed that an isocaloric healthy ND improved the lipid profile, low-grade inflammation, and ambulatory blood pressure among subjects with MetS (Uusitupa et al. 2013; Brader et al. 2014). No changes in glucose metabolism were observed since it may be difficult to improve glucose metabolism in established MetS without attendant weight loss and very distinct changes in the diet (Uusitupa et al. 2013).

The peripheral blood mononuclear cells (PBMCs) include monocytes and lymphocytes, which are cells central in inflammation. These cells circulate in the body and are exposed to nutrients, bioactive food components, and metabolic tissues. Alterations in gene expression levels in these cells may therefore reflect systemic health (Afman et al. 2014). It has been shown that long-term dietary intervention studies change the gene expression of inflammatory genes and genes involved in lipid metabolism (Myhrstad et al. 2014; van Dijk et al. 2012a; De Mello et al. 2009; Bouwens et al. 2009), suggesting that PMBCs are a good model system identifying early risk markers (Visvikis-Siest et al. 2007) and are sensitive to dietary changes.

Stress responses can be more informative than static homeostasis on nutrition-related health. An acute glucose load of a 2-h oral glucose tolerance test (OGTT) is such a stress response, monitors the ability of the body to respond to glucose intake, and is primarily used for addressing the degree of glucose tolerance and insulin resistance (van Ommen et al. 2009). Several studies have shown that an OGTT (Choi et al. 2012; Kempf et al. 2007; Aljada et al. 2006) as well as fat challenge tests (Bouwens et al. 2010; Cruz-Teno et al. 2012; van Dijk et al. 2012b; Myhrstad et al. 2011) modulate the gene expression of inflammatory genes in leucocytes and mononuclear cells. PBMCs also reflect the immune component of the white adipose tissue transcriptome after OGTT and after oral lipid tolerance test (O’Grada et al. 2014), and thus, changes in PBMC gene expression may act as biomarkers of metabolic health not only in the fasting state but also in the postprandial state (O’Grada et al. 2014).

Changes in glucose and lipid homeostasis by acute challenge tests are linked to inflammation. It has been shown in dietary intervention studies that the quality of diet affects OGTT response and improves insulin sensitivity and glucose tolerance in individuals with MetS (Laaksonen et al. 2005; Paniagua et al. 2007). However, no previous study has examined the long-term effect of a dietary intervention on the OGTT response using PBMCs and gene expression analysis.

The main aim of this sub-population of the SYSDIET study was to investigate if a long-term (18–24 weeks) healthy ND could modify the expression of inflammation and lipid metabolism-related genes in PBMCs during 2hOGTT in individuals with MetS.

## Results

### Characteristics of the subjects

**Fig. 1:**
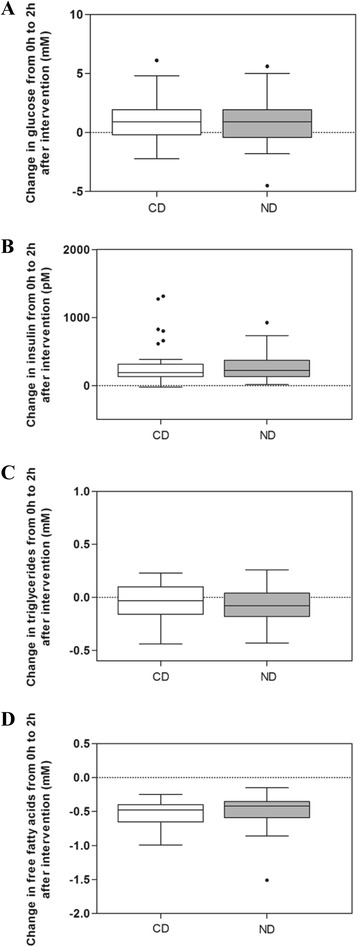
Changes in glucose, insulin, triglycerides, and free fatty acids from 0h to 2hOGTT after intervention. The effect of the healthy ND compared to the CD on changes in glucose (**a**), insulin (**b**), triglycerides (**c**), and free fatty acids (**d**) from 0 h to 2hOGTT after intervention. The effect of the independent variable study group is adjusted for changes in glucose, insulin, triglycerides, or free fatty acids at baseline, study centers, gender, age (log10 transformed), and body weight at the end of the study. *Box plots* show the medians with 25th and 75th percentiles. *Whiskers* express the 1.5 × interquartile range

At baseline, no differences were observed between the CD and ND groups related to age, BMI, serum lipids, glucose, insulin, circulating inflammation markers, lipid-lowering drugs, antihypertensive drugs, smoking, and MetS (Table 1). The change in glucose, insulin, triglycerides, and free fatty acids from 0h (fasting) to 2h (after OGTT) was not significantly different between the CD and ND groups (*P* = 0.330, *P* = 0.845, *P* = 0.196, and *P* = 0.681, respectively) (Fig. 1).

**Table 1:** Baseline characteristics of the participants

	Number	CD	Number	ND	*P*
Sex (female)	40	25 (63 %)	49	34 (69 %)	0.51
Age (year)	40	55.8 ± 7.8	49	54.4 ± 8.3	0.43
BMI (kg m^−2^)	40	31.9 ± 2.7	49	31.8 ± 3.1	0.90
Total cholesterol (mM)	40	5.3 ± 1.0	49	5.3 ± 1.0	0.91
LDL cholesterol (mM)	40	3.3 ± 0.9	49	3.2 ± 0.9	0.93
HDL cholesterol (mM)	40	1.3 ± 0.5	49	1.4 ± 0.3	0.51
Fasting triglycerides (mM)	40	1.5 ± 0.5	49	1.5 ± 0.7	0.84
Fasting glucose (mM)	40	5.8 ± 0.6	49	5.8 ± 0.6	0.46
Fasting insulin (pM)	40	59.5 (47.0–82.3)	49	55.0 (41.0–75.5)	0.43
IL1Ra (ng L^−1^)	40	308.7 (233.4–465.6)	49	203.8 (220.0–502.0)	0.96
IL1β (ng L^−1^)	39	0.12 (0.12–0.21)	49	0.12 (0.12–0.14)	0.44
IL6 (ng L^−1^)	40	1.3 (1.1–1.8)	49	1.3 (1.0–1.9)	0.76
IL10 (ng L^−1^)	39	0.9 (0.8–1.5)	49	0.8 (0.8–1.5)	0.32
sTNFRII (ng L^−1^)	40	1899.6 ± 415.4	49	1954.5 ± 461.1	0.56
hs-CRP (mg L^−1^)	40	1.5 (0.9–3.7)	49	1.5 (0.8–2.9)	0.68
HMW adiponectin (μg L^−1^)	40	3.6 (2.2–6.7)	49	4.0 (2.8–6.5)	0.36
Lipid-lowering drugs	40	13 (33 %)	49	12 (25 %)	0.48
Antihypertensive drugs	40	20 (50 %)	49	31 (63 %)	0.28
Smoking	40	6 (15 %)	49	4 (8 %)	0.34
Metabolic syndrome	40	34 (85 %)	49	42 (86 %)	1.00

Values are expressed as means ± SDs, medians (25th–75th percentiles), or numbers (%)

*CD* control diet, *ND* healthy Nordic diet, *BMI* body mass index, *LDL* low density lipoprotein, *HDL* high-density lipoprotein, *IL1Ra* interleukin-1 receptor antagonist, *IL1β* interleukin-1 beta, *IL6* interleukin 6, *IL10* interleukin 10, *sTNFRII* tumor necrosis factor receptor 2, *hs*-*CRP* high-sensitivity C-reactive protein, *HMW adiponectin* human high molecular weight adiponectin

### Dietary data

The dietary intake of this sub-population is shown in Table 2. The results are in line with the original analysis with the whole SYSDIET study population (Uusitupa et al. 2013). The intake of polyunsaturated fatty acids was higher and of saturated fatty acids lower in the ND compared to the CD group. Further, α-linolenic acid, fiber, β-carotene, vitamin C, vitamin E, folate, potassium, and magnesium intake were higher in the ND versus the CD group.

**Table 2:** Dietary intake in the CD and healthy ND group at baseline and end of intervention

	CD (*n* = 40)		ND (*n* = 47)		Regression coefficient β (95 % CI)		
	Baseline	End	Baseline	End	Unadjusted	Adjusted	*P* ^a^
Energy, kJ	8074 ± 2173	8301 ± 1542	8077 ± 1757	8537 ± 1791	235 (−484 to 954)	297 (−218 to 813)	0.254
Protein, E%	17.2 ± 2.4	17.0 ± 2.3	16.7 ± 2.7	16.8 ± 2.3	−0.1 (−1.1 to 0.9)	0.1 (−0.8 to 1.0)	0.772
Carbohydrate, E%	46.4 ± 2.3	43.2 ± 7.0	45.1 ± 5.8	45.5 ± 5.2	2.3 (0.6 to 4.5)	2.5 (0.5 to 4.4)	0.013
Sucrose, g	40.1 ± 17.3	34.8 ± 15.4	41.2 ± 15.2	37.6 ± 15.6	2.8 (−3.9 to 9.4)	1.8 (−4.0 to 7.6)	0.541
Fat, E%	32.1 ± 6.2	35.7 ± 5.1	32.5 ± 7.3	32.9 ± 5.1	−2.8 (−4.9 to − 0.6)	−2.9 (−5.0 to − 0.9)	0.005
SFA, E%	13.0 ± 3.2	15.3 ± 2.9	13.2 ± 3.5	10.8 ± 2.3	−4.5 (−5.6 to − 3.4)	−4.8 (−5.8 to − 3.7)	<0.001
MUFA, E%	11.5 ± 2.3	12.8 ± 2.0	11.6 ± 2.8	12.7 ± 2.2	−0.1 (−1.0 to 0.8)	−0.1 (−0.9 to 0.7)	0.864
PUFA, E%	4.7 ± 1.6	4.5 ± 1.1	4.9 ± 1.4	6.9 ± 1.5	2.5 (1.9 to 3.0)	2.5 (1.9 to 3.0)	<0.001
Linoleic acid, g	7.3 ± 2.4	8.2 ± 2.8	8.0 ± 3.0	8.7 ± 3.9	0.5 (−0.9 to 2.0)	0.5 (−0.9 to 1.9)	0.517
α-Linolenic acid, g	1.2 ± 0.6	1.4 ± 0.7	1.2 ± 0.6	2.0 ± 1.4	0.7 (0.2 to 1.2)	0.7 (0.4 to 1.1)	<0.001
Fiber, g	21.2 ± 6.5	16.4 ± 4.9	21.7 ± 7.2	36 ± 10.1	19.6 (16.2 to 23.1)	19.5 (16.2 to 22.8)	<0.001
Cholesterol, mg	268 ± 127	283 ± 118	254 ± 102	214 ± 74	−69 (−111 to − 28)	−58 (−90 to − 25)	0.001
Salt, g	7.2 ± 2.9	7.0 ± 2.0	7.1 ± 2.2	6.5 ± 2.4	−0.4 (−1.4 to 0.5)	−0.3 (−1.1 to 0.5)	0.478
β-Carotene, mg	2561 ± 2090	1733 ± 1173	2449 ± 1891	2987 ± 1857	1254 (578 to 1930)	1213 (543 to 1884)	0.001
Vitamin C, mg	120 ± 100	66 ± 32	112 ± 59	138 ± 50	72 (54 to 90)	72 (54 to 91)	<0.001
Vitamin E, mg	8.9 ± 3.4	8.2 ± 2.2	9.5 ± 3.4	13.6 ± 3.0	5.3 (4.2 to 6.5)	5.2 (4.1 to 6.2)	<0.001
Folate, mg	251 ± 76	226 ± 63	271 ± 83	343 ± 172	117 (60 to 174)	104 (51 to 158)	<0.001
Sodium, mg	2855 ± 1151	2722 ± 78	2813 ± 878	2654 ± 980	−68 (−451 to 314)	−27 (−337 to 284)	0.865
Potassium, mg	3711 ± 1175	3276 ± 933	3626 ± 975	4017 ± 910	742 (348 to 1135)	767 (482 to 1053)	<0.001
Magnesium, mg	359 ± 111	309 ± 82	370 ± 101	421 ± 101	112 (73 to 152)	106 (79 to 132)	<0.001
Calcium, mg	1006 ± 411	951 ± 383	967 ± 370	997 ± 310	46 (−102 to 193)	60 (−43 to 163)	0.250
Alcohol, E%	2.1 ± 2.8	3.0 ± 3.3	3.0 ± 4.1	1.8 ± 2.9	−1.2 (−2.5 to 0.1)	−1.2 (−2.3 to − 0.1)	0.036

Values are means ± SDs

*CD* control diet, *ND* healthy Nordic diet, *E %* percentage of energy, *SFA* saturated fatty acids, *MUFA* monounsaturated fatty acids, *PUFA* polyunsaturated fatty acids

^a^With linear multivariable regression analysis, the effect of the independent variable “study group” adjusted for dietary data at baseline, study center, gender, log10-transformed age, and body weight at the end of the intervention was assessed. The regression coefficient expresses the mean difference between the groups, unadjusted and adjusted. The CD and the ND groups did not differ from each other at baseline (*P* > 0.05)

### Changes in 2hOGTT gene expression response at baseline

Since it is well known that glucose uptake inhibits pyruvate dehydrogenase kinase, isozyme 4 (PDK4), *PDK4* mRNA expression in PBMCs was used as a positive control for the 2hOGTT response. A significant down-regulation of *PDK4* mRNA expression from 0h (fasting) to 2h (after OGTT) was observed in the whole study population (*q* < 0.0001) (Additional file 1). Transcript levels of several inflammatory and lipid metabolism-related genes were regulated after the OGTT (up-regulation with fold changes between 1.11 and 1.36 and down-regulation with fold changes between 0.72 and 0.93 (*q* < 0.05)) (Additional file 1).

### Changes in 2hOGTT gene expression response after dietary intervention

**Fig. 2:**
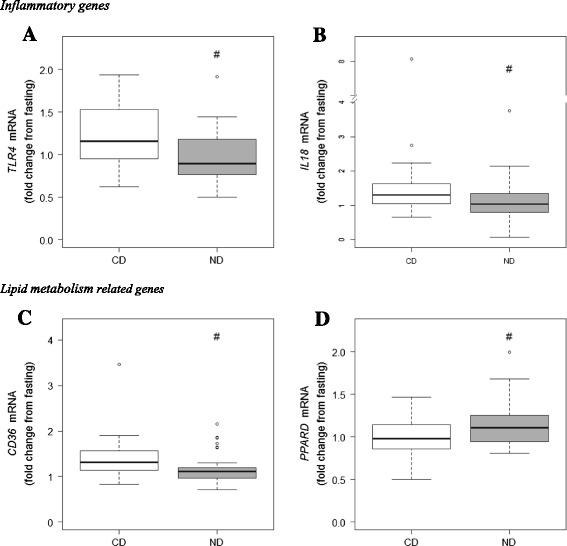
Effect of ND compared to CD on gene expression changes after a 2hOGTT. The effect of the healthy ND compared to the CD on *TLR4* mRNA expression (**a**), *IL18* mRNA expression (**b**), *CD36* mRNA expression (**c**), and *PPARD* mRNA expression (**d**) presented as fold change (2^−ΔCt^ of 2hOGTT/2^−ΔCt^ of 0hOGTT) in the healthy ND group (*n* = 42–49) and in the CD group (*n* = 32–40) after intervention. Fold changes (2^−∆ΔCt^) are normalized for the reference gene *TBP* and fasting values (0hOGTT). The *box plots* show the 2^−∆ΔCt^ values at the end of the intervention. *Box plots* show the medians with 25th and 75th percentiles. Whiskers express the 1.5 × interquartile range. The *number sign* is a *q* value of 0.042. The *q* values indicate the effect of the ND compared to the CD on gene expression changes after a 2hOGTT. The effect of the independent variable study group is adjusted for fold change at baseline (log2 transformed) and study centers. A *q* value <0.05 (FDR < 5 %) was considered significant

To study if the healthy ND could change the 2hOGTT gene expression response in PBMCs, we conducted a linear multiple regression analysis adjusting for changes at baseline and differences in the study centers. Among the 44 genes, the healthy ND significantly down-regulated the expressions of toll-like receptor 4 (*TLR4*) (*β* = −0.33, *q* = 0.042), interleukin 18 (*IL18*) (*β* = −0.73, *q* = 0.042), and *CD36* (*β* = −0.23, *q* = 0.042) compared to the CD after the OGTT (Table 3 and Fig. 2a–c). In contrast, a healthy ND significantly up-regulated the expression of peroxisome proliferator-activated receptor delta (*PPARD*) (*β* = 0.21, *q* = 0.042) compared to the CD after the OGTT (Table 3 and Fig. 2d).

**Table 3:** Effect of the healthy ND compared to the CD on gene expression changes after 2hOGTT

	Number	Group effect (regression coefficient β)	95 % CI for β	*q* values
Inflammatory genes				
*CCL2*				
Unadjusted	78	0.12	−0.19–0.43	0.64
Adjusted	78	0.12	−0.20–0.42	0.69
*CCL5*				
Unadjusted	85	−0.002	−0.14–0.14	0.98
Adjusted	85	−0.002	−0.14–0.14	0.98
*CCR2*				
Unadjusted	76	−0.06	−0.27–0.15	0.72
Adjusted	76	−0.07	−0.28–0.14	0.69
*CCR4*				
Unadjusted	77	−0.07	−0.23–0.10	0.64
Adjusted	77	−0.06	−0.22–0.11	0.69
*CD40*				
Unadjusted	87	−0.14	−0.37–0.10	0.46
Adjusted	87	−0.03	−0.25–0.19	0.88
*CD40LG*				
Unadjusted	87	−0.09	−0.22–0.04	0.42
Adjusted	87	−0.08	−0.21–0.06	0.47
*CXCR2*				
Unadjusted	86	−0.27	−0.47–(−0.07)	0.05
Adjusted	86	−0.25	−0.45–(−0.05)	0.09
*ICAM1*				
Unadjusted	84	−0.02	−0.22–0.19	0.89
Adjusted	84	0.02	−0.19–0.22	0.93
*IFNG*				
Unadjusted	86	−0.29	−0.53–(−0.05)	0.09
Adjusted	86	−0.27	−0.51–(−0.02)	0.13
*IKBKB*				
Unadjusted	83	−0.24	−0.41–(−0.06)	0.05
Adjusted	83	−0.24	−0.42–(−0.06)	0.08
*IL18*				
Unadjusted	72	−0.60	−1.01–(−0.18)	0.05
Adjusted	72	−0.73	−1.20–(−0.26)	*0.042*
*IL1B*				
Unadjusted	85	−0.17	−0.43–0.10	0.42
Adjusted	85	−0.16	−0.41–0.09	0.45
*IL1RN*				
Unadjusted	87	−0.10	−0.26–0.06	0.42
Adjusted	87	−0.10	−0.26–0.06	0.47
*IL23A*				
Unadjusted	87	0.15	0.00–0.31	0.17
Adjusted	87	0.14	−0.01–0.30	0.20
*IL23R*				
Unadjusted	88	−0.06	−0.36–0.24	0.78
Adjusted	88	−0.05	−0.34–0.25	0.88
*IL6*				
Unadjusted	86	−0.18	−0.45–0.09	0.42
Adjusted	86	−0.19	−0.46–0.09	0.43
*IL8*				
Unadjusted	83	−0.50	−1.09–0.09	0.30
Adjusted	83	−0.53	−1.12–0.05	0.22
*MMP9*				
Unadjusted	71	−0.09	−0.52–0.34	0.78
Adjusted	71	−0.12	−0.57–0.33	0.74
*NFKBIA*				
Unadjusted	87	0.09	−0.03–0.22	0.37
Adjusted	87	0.10	−0.03–0.22	0.34
*OLR1*				
Unadjusted	ND	ND	ND	ND
Adjusted	ND	ND	ND	ND
*PDGFA*				
Unadjusted	64	0.21	−0.06–0.48	0.37
Adjusted	64	0.29	0.00–0.57	0.17
*PDGFB*				
Unadjusted	77	0.24	0.03–0.45	0.12
Adjusted	77	0.24	0.03–0.45	0.11
*PDK4*				
Unadjusted	88	−0.14	−0.41–0.13	0.55
Adjusted	88	−0.08	−0.34–0.18	0.70
*RELA*				
Unadjusted	83	−0.06	−0.21–0.10	0.64
Adjusted	83	−0.10	−0.25–0.06	0.45
*TGFB2*				
Unadjusted	75	0.12	−0.15–0.39	0.64
Adjusted	75	0.10	−0.18–0.38	0.69
*TLR4*				
Unadjusted	85	−0.36	−0.54–(−0.18)	*0.008*
Adjusted	85	−0.33	−0.52–(−0.15)	*0.042*
*TNF*				
Unadjusted	86	−0.09	−0.23–0.05	0.42
Adjusted	86	−0.09	−0.23–0.05	0.45
*TNFRSF1A*				
Unadjusted	88	−0.24	−0.41–(−0.07)	0.05
Adjusted	88	−0.22	−0.40–(−0.04)	0.09
*TNFRSF1B*				
Unadjusted	88	−0.11	−0.29–0.08	0.46
Adjusted	88	−0.10	−0.28–0.08	0.47
Lipid metabolism-related genes				
*ABCA1*				
Unadjusted	72	−0.12	−0.39–0.16	0.64
Adjusted	72	−0.12	−0.39–0.15	0.64
*ABCG1*				
Unadjusted	81	0.09	−0.14–0.32	0.64
Adjusted	81	0.11	−0.12–0.34	0.60
*CD36*				
Unadjusted	85	−0.24	−0.40–(−0.07)	0.05
Adjusted	85	−0.23	−0.39–(−0.08)	*0.042*
*CPT1A*				
Unadjusted	78	0.05	−0.17–0.26	0.78
Adjusted	78	0.05	−0.27–0.27	0.80
*CPT1B*				
Unadjusted	82	−0.26	−0.49–(−0.02)	0.12
Adjusted	82	−0.26	−0.49–(−0.03)	0.13
*CRAT*				
Unadjusted	84	0.01	−0.13–0.16	0.89
Adjusted	84	0.02	−0.13–0.17	0.88
*HMGCR*				
Unadjusted	86	0.04	−0.12–0.19	0.78
Adjusted	86	0.04	−0.12–0.20	0.74
*LDLR*				
Unadjusted	73	0.24	0.04–0.45	0.10
Adjusted	73	0.25	0.04–0.46	0.09
*LIPE*				
Unadjusted	ND	ND	ND	ND
Adjusted	ND	ND	ND	ND
*NAMPT*				
Unadjusted	80	−0.07	−0.25–0.12	0.64
Adjusted	80	−0.06	−0.25–0.12	0.69
*PLIN2*				
Unadjusted	80	0.02	−0.18–0.21	0.89
Adjusted	80	0.004	−0.20–0.20	0.98
*PPARA*				
Unadjusted	87	0.03	−0.15–0.21	0.81
Adjusted	87	0.02	−0.16–0.20	0.88
*PPARD*				
Unadjusted	87	0.22	0.09–0.36	*0.021*
Adjusted	87	0.21	0.08–0.35	*0.042*
*SREBF1*				
Unadjusted	81	−0.15	−0.69–0.39	0.74
Adjusted	81	−0.24	−0.77–0.28	0.61
*UCP2*				
Unadjusted	84	0.16	−0.04–0.36	0.32
Adjusted	84	0.17	−0.02–0.36	0.23

Adjusted models: The effect of the independent variable “study group” is adjusted for fold change at baseline (log2 transformed) and study center. It should be noted that the regression coefficient expresses the mean difference between the groups, unadjusted and adjusted. A *q* value <0.05 (FDR < 5 %) were considered significant

*ND* not detected

## Discussion

In the present study, we found that the healthy ND modulated the mRNA levels of *TLR4*, *IL18*, *CD36*, and *PPARD* differently after the OGTT compared to the CD. We also showed that several genes related to inflammation and lipid metabolism were significantly modulated by an OGTT in PBMCs of subjects with MetS at baseline.

Immune response and lipid metabolism are closely linked in metabolic diseases, and alterations in these responses after a food challenge may play an important role in the prevention and early detection of diseases (van Ommen et al. 2009). The healthy ND down-regulated the expression of *TLR4* compared to the CD group after the OGTT. TLR4 is involved in the pro-inflammatory response by regulating nuclear factor kappa B (NFκB) activity (Doyle and O’Neill 2006) and is a key regulator of immune response. Previously, it has been reported that an increased *TLR4* mRNA expression in monocytes in individuals with MetS compared to healthy controls (Hardy et al. 2013) and down-regulation of the *TLR4* mRNA expression by weight loss are associated with improvement of insulin sensitivity in the individuals with MetS (De Mello et al. 2008). This would indicate that the ND compared to the CD may be less inflammatory and may lead to improvement of insulin sensitivity via *TLR4* down-regulation in the present study. We were, however, not able to show any improvement in insulin sensitivity or fasting glucose or insulin concentrations in the main SYSDIET trial (Uusitupa et al. 2013), suggesting that the number of subjects in the original study was too low to see an effect. Anyhow, it may be speculated that the use of PBMC gene expression analysis could serve as a more sensitive model system than measurement of circulating markers of glucose metabolism.

Interestingly, we observed that the healthy ND reduced the *IL18* mRNA expression after the OGTT compared to CD. IL18 is a pro-inflammatory cytokine shown to be a strong predictor of cardiovascular events in elderly men with MetS, and the effect is stronger with elevated fasting glucose (Troseid et al. 2009). The mRNA expression of *IL18* is also increased in obese individuals, and this increase is correlated with insulin resistance (Ahmad et al. 2013). Thus, the down-regulation of *IL18* mRNA after the OGTT by healthy ND in the present study may indicate the impact of the ND on disease development among the individuals with MetS.

CD36 is a scavenger receptor involved in lipid uptake and foam cell formation in macrophages (Silverstein 2009). Intracellular lipids taken up by CD36 activate TLR4, which generate pro-inflammatory signals by activating NFκB (Fessler et al. 2009). A blockage of TLR4 and CD36 in human macrophages reduced secretion of IL1β, IL6, and IL8 and the subsequent foam cell formation (Chavez-Sanchez et al. 2014). We found a down-regulation of *CD36* mRNA transcript level after the OGTT in the healthy ND group compared to the CD group. We could speculate that the beneficial effects of ND may be executed also via reduction of the foam cell formation and inflammation indicated by down-regulation of the *CD36* mRNA expression in the postprandial state. In contrast, the *PPARD* gene transcript was increased after the OGTT challenge in the ND group compared to the CD group. *PPARD* is expressed in several tissues in the body, including macrophages. The expression of *PPARD* genes regulates lipid metabolism and glucose homeostasis, increases fatty acid oxidation, and decreases inflammation as well as platelet activation (Monsalve et al. 2013). We have previously shown that obese subjects at risk had reduced PBMC gene expression of *PPARD* compared with metabolically healthy obese and control subjects (Telle-Hansen et al. 2013), and PPARD activation improves multiple metabolic disorders (especially blood lipids) in obese subjects (Riserus et al. 2008).

In accordance to other studies, the expression of PDK4 was down-regulated (Zhang et al. 2014), and several pro-inflammatory genes (*TNF*, *TGFB2*, *CXCR2*, *CD40LG*, *IL1RN*, *CCR2*, *IL23R*, and *MMP9*) and lipid metabolism related genes (*CD36*, *ABCG1* and *ABCA1*) were up-regulated, after the OGTT in the whole study population, confirming the use of PBMC gene expression analysis as a model system to detect metabolic responses after an OGTT (Aljada et al. 2006; Aljada et al. 2004; Griffin et al. 2001). The mRNA level of *CPT1A* was down-regulated during 2hOGTT. Since CPT1 is involved in oxidation of fatty acids, and the oxidation is suppressed in the presence of an adequate glucose supply (Bonnefont et al. 2004), the reduced expression of *CPT1* during 2hOGTT may be explained by increased glucose oxidation and decreased fatty acid oxidation.

The strength of this study is the relatively high number of subjects, and to the best of our knowledge, the current dietary intervention study is the first one to use PBMC gene expression as a tool to examine if diet can modify the OGTT response. We used a well-characterized glucose-regulated gene as a positive control to ensure that changes in mRNA level could be measured 2h after OGTT. The limitation of the study is that we cannot differentiate any specific food components responsible for the effect on the change in 2hOGTT response since we did not focus the intervention on single nutrients but on the whole diet. Our primary aim was however to study the effects of the whole diet, since this approach is closer to real-life situations.

## Conclusions

We show that the long-term intake of a healthy ND down-regulates genes involved in inflammation and lipid metabolism in individuals at risk for metabolic diseases and thereby may reduce this unfavorable postprandial response. The results need to be confirmed by further human intervention studies, preferably with meal challenges. In addition, experimental models (e.g., ex vivo cell models or animal model) should be employed to extend our biological and clinical understanding of the data presented here.

## Methods

### Study design and subjects

The study design and participants have been described in detail elsewhere (Uusitupa et al. 2013). In short, this study was a randomized controlled multicenter study performed in six centers within the Nordic countries [Kuopio and Oulu (Finland), Lund and Uppsala (Sweden), Aarhus (Denmark), and Reykjavik (Iceland)]. The participants were randomized after a 4-week run-in period with habitual diet into a healthy ND group or a CD group for 18–24 weeks. The composition of the diets has been described in detail elsewhere (Uusitupa et al. 2013). The main differences between the diets at the nutrient level were the amount of dietary fiber and salt and the quality of dietary fat. Both the ND and the CD were isocaloric based on the evaluation of the habitual diet calculated from a 4-day food record during the run-in period. The Nordic Nutrition Recommendations (NNR) formed the basis of the ND, and the main emphasis was on food items such as whole-grain products, abundant use of berries, fruit and vegetables, rapeseed oil, three fish meals per week, low-fat dairy products, and avoidance of sugar-sweetened products. The subjects in the CD consumed a diet in accordance to the mean nutrient intake in the Nordic countries. Key products were provided to the study participants in both groups. The study participants were advised to keep body weight and physical activity constant and not to change their smoking and drinking habits or drug treatment during the study. All study participants provided their written informed consent, and local ethics committees of all the participating centers approved the study protocol.

Altogether, 309 individuals were originally contacted and screened at the study clinics, and 213 were randomized as described earlier (Uusitupa et al. 2013). Ninety-six individuals in the ND group and 70 in the CD group completed the trial (Uusitupa et al. 2013). The inclusion criteria were age 30–65 years, BMI 27–38 kg m^−2^, and two other of the International Diabetes Federation (IDF) criteria for MetS (Alberti et al. 2009). Antihypertensive and lipid-lowering medication, as well as inhaled corticosteroids, were allowed but without dosage changes during the trial. The main exclusion criteria included any chronic disease and condition, which could hamper the adherence to the dietary intervention protocol, poor compliance, chronic liver, thyroid and kidney diseases, alcohol abuse (>40 g per day), diabetes, fasting triglycerides >3.0 mM, total cholesterol >6.5 mM, and blood pressure >160/100 mmHg. A few study participants with triglycerides between 3 and 4 mM and with BMI between 38 and <40 kg m^−2^ were, however, included in the main study population.

**Fig. 3:**
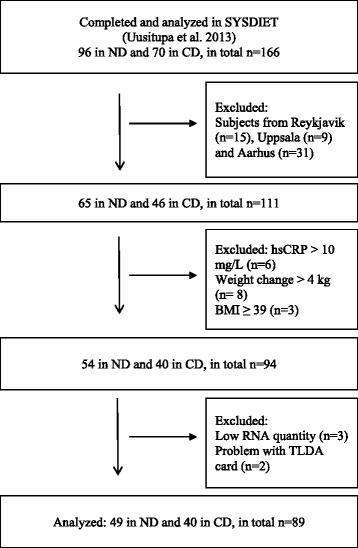
Flow chart of the participants

In this present sub-study of the SYSDIET trial, we included a total of 94 subjects (*n* = 54 in ND and *n* = 40 in CD) out of the 166 subjects who completed the SYSDIET study (Fig. 3). We excluded subjects from Aarhus (*n* = 31), Uppsala (*n* = 9), and Reykjavik (*n* = 15), because the Aarhus study center did not collect PBMC samples, and the number of PBMC samples was limited from Uppsala (*n* = 9) and Reykjavik (*n* = 5). So, by excluding these two centers, we reduced variance. We also excluded subjects with high-sensitivity C-reactive protein (hs-CRP) concentration higher than 10 mg L^−1^ at baseline or after the intervention (*n* = 6), baseline BMI above 39 kg m^−2^ (*n* = 3), and body weight change more than 4 kg during the intervention (*n* = 8). Since five subjects were excluded from the analysis due to low quantity of RNA (*n* = 3) or problems with the TaqMan Array Micro Fluidic Cards (*n* = 2), we analyzed data from 89 subjects (*n* = 49 in ND and *n* = 40 in CD) (Fig. 3).

### Clinical and biochemical measurements

Procedures regarding the clinical and biochemical measurements have been described previously (Uusitupa et al. 2013). In short, subjects were examined in the morning after overnight fasting. Anthropometric measurements were performed locally according to the standard operational procedures. Concentrations of plasma glucose, cholesterol, and triglycerides were analyzed locally in the centers using routine methodology. Blood samples to measure cytokines and adipokines from all the study centers were analyzed in the University of Eastern Finland and Kuopio University Hospital, Finland. Plasma insulin was analyzed in the Aarhus University Hospital, Denmark, using routine automated clinical chemistry analyzers.

### Standard 2hOGTT

A standard 2hOGTT (75 g D-glucose) was performed after an overnight fast at baseline and at the end of the intervention. Blood samples for PBMC isolation were taken at the time points 0 and 120 min.

### PBMCs, RNA isolation, cDNA synthesis, and qPCR

After blood collection, PBMCs were isolated at baseline and at the end of the intervention at time points 0 and 120 min by using the BD Vacutainer Cell Preparation tubes according to the manufacturer’s instructions (Becton, Dickinson San Jose, CA, USA) and stored as pellets at −80 °C for further analysis. Total RNA isolation was performed centrally at the Karolinska Institute (Stockholm, Sweden). The total RNA was isolated using the RNeasy Mini Kit according to the manufacturer’s instructions (Qiagen, Valencia, CA, USA). RNA quantity and quality measurements were performed using a Nanodrop ND-1000 Spectrophotometer (Thermo Fisher Scientific, Gothenburg, Sweden) and Agilent 2100 Bioanalyzer (Agilent Technologies, Santa Clara, CA, USA), respectively. RNA from all samples was reverse transcribed by a high-capacity cDNA reverse transcription kit (Applied Biosystems, Foster City, CA, USA). Quantitative real-time polymerase chain reaction (qPCR) was performed on an ABI PRISM 7900HT (Applied Biosystems) using TaqMan Array Micro Fluidic Cards (Applied Biosystems). The target genes are shown in the Additional file 1. Primer sequences are commercially available (Applied Biosystems) and can be provided upon request. The selection of target genes was primarily based on previous long-term and short-term dietary intervention studies where PBMC gene expression of inflammatory and lipid metabolism genes was modulated or associated with features of MetS or metabolic risk factors for T2DM and CVD (De Mello et al. 2009; Bouwens et al. 2009; Jones et al. 2011; Kaminski et al. 1993) and on results from our own studies (Myhrstad et al. 2011; Telle-Hansen et al. 2013).

The relative mRNA level for each transcript was calculated by the ∆Δ cycle threshold (Ct) method (Livak and Schmittgen 2001). TATA-box binding protein (*TBP*) was used as reference gene for normalization. Briefly, the Ct values of each target gene were normalized to the Ct values of the *TBP* (=ΔCt). The fold change in mRNA gene expression from *TBP* was calculated at fasting (0h) and after (2h) OGTT (2^−ΔCt^) at baseline and at the end of the intervention. The fold change in mRNA gene expression from fasting was calculated as 2^−∆ΔCt^ at baseline and at the end of the intervention, as 2^−ΔCt^_2hOGTT_ was divided by 2^−ΔCt^_0hOGTT_.

### Statistical analysis

For baseline characteristic comparisons, we used independent *t* test to test the difference between means, Mann-Whitney *U* test to test the difference between medians and chi-square test to test the difference between categorical variables. Power calculations (alpha <0.05, beta >0.8) were carried out on serum cholesterol, fasting glucose, and insulin (Uusitupa et al. 2013).

Linear multivariable regression analyses were used to test the independent effect of the study groups on the change in glucose, insulin, triglycerides, and free fatty acids from 0h (fasting) to 2h (after OGTT) at the end of the intervention as well as on the dependent dietary intake variables at the end of the intervention. The effect of the independent variable study group was adjusted for the corresponding baseline variable, study centers, gender, log10-transformed age, and body weight at the end of the intervention. Changes in gene expression from 0h to 2hOGTT within the whole study population were tested with Wilcoxon signed rank test (2^−ΔCt^). Data are given as the median (25–75th percentiles). Linear multivariable regression analyses were also carried out to test the independent effect of the study groups on the dependent variable fold change at the end of the intervention. The analyses were adjusted for the independent variables fold change at baseline and study center. In the presentation, β denotes the regression coefficient of the treatment group. Fold changes at baseline and at the end of the intervention (2^−∆ΔCt^) were log2 transformed before the analyses to improve normality. To account for multiple testing, we applied false discovery rate (FDR) analysis and *q* < 0.05 (FDR < 5 %) was considered significant. Calculations were performed using IBM SPSS Statistics version 20 (Armonk, NY, USA) and R version 3.2.0.

## Additional file

Additional file 1: mRNA level at fasting (0hOGTT) and after 2hOGTT and fold change from fasting in the whole study population at baseline. Data for 0hOGTT and 2hOGTT is given as 2^−∆Ct^ (normalized for *TBP*). Data for fold change is given as 2^−∆∆Ct^ (normalized for *TBP* and 0hOGTT values). All values are presented as medians with 25th–75th percentiles. (DOCX 21.4 kb)

## Abbreviations

ABCA1: ATP-binding cassette, sub-family A (ABC1), member 1
ABCG1: ATP-binding cassette, sub-family G, member 1
CCL2: chemokine (C-C motif) ligand 2
CCL5: chemokine (C-C motif) ligand 5
CCR2: chemokine (C-C motif) receptor 2
CCR4: chemokine (C-C motif) receptor 4
CD: control diet
CD36: CD36 molecule (thrombospondin receptor)
CD40: CD40 molecule
CD40LG: CD40 ligand
CPT1A: carnitine palmitoyltransferase 1A
CPT1B: carnitine palmitoyltransferase 1B
CRAT: carnitine O-acetyltransferase
CXCR2: chemokine (C-X-C motif) receptor 2
CVD: cardiovascular diseases
FDR: false discovery rate
HMGCR: 3-hydroxy-3-methylglutaryl-CoA reductase
ICAM1: intercellular adhesion molecule 1
IFNG: interferon, gamma
IKBKB: inhibitor of kappa light polypeptide gene enhancer in B cells, kinase beta
IL18: interleukin 18
IL1B: interleukin 1, beta
IL1RN: interleukin 1 receptor antagonist
IL23A: interleukin 23, alpha subunit p19
IL23R: interleukin 23, receptor
IL6: interleukin 6
IL8: interleukin 8
LDLR: low-density lipoprotein receptor
LIPE: lipase, hormone-sensitive
MetS: metabolic syndrome
MMP9: matrix metallopeptidase 9
NAMPT: nicotinamide phosphoribosyltransferase
ND: Nordic diet
NFKBIA: nuclear factor of kappa light polypeptide gene enhancer in B cells inhibitor, alpha
OLR1: oxidized low density lipoprotein (lectin-like) receptor 1
PBMCs: peripheral blood mononuclear cells
PDGFA: platelet-derived growth factor alpha polypeptide
PDGFB: platelet-derived growth factor beta polypeptide
PDK4: pyruvate dehydrogenase kinase, isozyme 4
PLIN2: perilipin 2
PPARA: peroxisome proliferator-activated receptor alpha
PPARD: peroxisome proliferator-activated receptor delta
RELA: v-rel reticuloendotheliosis viral oncogene homologue A
SREBF1: sterol regulatory element binding transcription factor 1
SYSDIET: Systems Biology in Controlled Dietary Interventions and Cohort Studies
T2DM: type 2 diabetes mellitus
TGFB2: transforming growth factor beta 2
TLR4: toll-like receptor 4
TNF: tumor necrosis factor
TNFRSF1A: tumor necrosis factor receptor superfamily member 1A
TNFRSF1B: tumor necrosis factor receptor superfamily member 1B
UCP2: uncoupling protein 2
