# Supplementary material for: Effects of a healthy Nordic diet on gene expression changes in peripheral blood mononuclear cells in response to an oral glucose tolerance test in subjects with metabolic syndrome: a SYSDIET sub-study
Source: Genes Nutr. 2016 Mar 17;11:3. doi: 10.1186/s12263-016-0521-4 (PMC4959556; doi:10.1186/s12263-016-0521-4)
Supplement: Additional file 1: — mRNA level at fasting (0hOGTT) and after 2hOGTT and fold change from fasting in the whole study population at baseline. Data for 0hOGTT and 2hOGTT is given as 2−∆Ct (normalized for TBP). Data for fold change is given as 2−∆∆Ct (normalized for TBP and 0hOGTT values). All values are presented as medians with 25th–75th percentiles. (DOCX 21.4 kb) [file 12263_2016_521_MOESM1_ESM.docx]

**Additional file 1:** mRNA level at fasting (0 h OGTT), after 2 h OGTT and fold change from fasting in the whole study population at baseline. Data for 0 h OGTT and 2 h OGTT is given as 2^-∆Ct^ (normalized for *TBP*). Data for fold change is given as 2^-∆∆Ct^ (normalized for *TBP* and 0h OGTT values). All values are presented as medians with 25^th^ -75^th^ percentiles.

| Target gene | n | 0h OGTT | 2h OGTT | Fold change  (from 0h OGTT values) | q-values^a^ |
| --- | --- | --- | --- | --- | --- |
| **Inflammation** |  |  |  |  |  |
| *CCL2* | 84 | 0.04 (0.02 - 0.05) | 0.04 (0.02 - 0.06) | 1.12 (0.78 - 1.65) | 0.15 |
| *CCL5* | 86 | 79.10 (59.66 - 98.55) | 70.42 (59.67 - 92.99) | 0.96 (0.82 - 1.11) | 0.20 |
| *CCR2* | 80 | 6.07 (4.58 - 7.37) | 6.74 (5.91 - 8.55) | 1.23 (0.92 - 1.57) | **0.0016** |
| *CCR4* | 79 | 2.38 (1.98 - 3.15) | 2.58 (2.04 - 3.19) | 1.04 (0.80 - 1.30) | 0.70 |
| *CD40* | 87 | 0.79 (0.62 - 0.96) | 0.80 (0.66 - 1.05) | 1.02 (0.88 - 1.24) | 0.64 |
| *CD40LG* | 87 | 1.28 (1.05 - 1.54) | 1.51 (1.20 - 1.68) | 1.18 (1.00 - 1.32) | **<0.0001** |
| *CXCR2* | 86 | 1.00 (0.87 - 1.27) | 1.16 (0.89 - 1.44) | 1.16 (0.94 - 1.42) | **0.0012** |
| *ICAM1* | 85 | 1.11 (0.91 - 1.40) | 1.02 (0.82 - 1.14) | 0.93 (0.68 - 1.16) | **0.0188** |
| *IFNG* | 88 | 0.16 (0.11 - 0.23) | 0.17 (0.13 - 0.23) | 1.05 (0.87 - 1.30) | 0.31 |
| *IKBKB* | 85 | 3.76 (3.19 - 4.14) | 3.74 (3.31 - 4.35) | 1.02 (0.86 - 1.18) | 0.78 |
| *IL18* | 81 | 0.19 (0.15 - 0.28) | 0.19 (0.15 - 0.23) | 0.97 (0.66 - 1.25) | 0.15 |
| *IL1B* | 85 | 0.50 (0.42 - 0.66) | 0.54 (0.41 - 0.73) | 0.97 (0.83 - 1.41) | 0.49 |
| *IL1RN* | 87 | 1.56 (1.28 - 1.90) | 1.84 (1.54 - 2.30) | 1.21 (0.99 - 1.40) | **<0.0001** |
| *IL23A* | 88 | 0.29 (0.22 - 0.34) | 0.28 (0.24 - 0.33) | 1.04 (0.81 - 1.30) | 0.49 |
| *IL23R* | 88 | 0.02 (0.01 - 0.03) | 0.02 (0.02 - 0.04) | 1.27 (1.00 - 1.75) | **<0.0001** |
| *IL6* | 86 | 0.02 (0.01 - 0.03) | 0.02 (0.01 - 0.03) | 0.86 (0.66 - 1.12) | **0.0027** |
| *IL8* | 85 | 0.02 (0.01 - 0.05) | 0.03 (0.02 - 0.06) | 1.31 (0.62 - 2.26) | 0.18 |
| *MMP9* | 80 | 0.05 (0.03 - 0.09) | 0.07 (0.05 - 0.10) | 1.36 (0.83 - 2.60) | **0.0049** |
| *NFKBIA* | 87 | 11.58 (10.09 - 13.49) | 10.88 (9.53 - 12.10) | 0.91 (0.80 - 1.03) | **0.0003** |
| *OLR1* | ND | ND | ND | ND | ND |
| *PDGFA* | 76 | 1.62 (0.94 - 2.11) | 1.45 (0.98 - 2.34) | 1.04 (0.80 - 1.34) | 0.64 |
| *PDGFB* | 82 | 0.79 (0.60 - 1.03) | 0.71 (0.57 - 0.95) | 0.93 (0.76 - 1.20) | 0.49 |
| *PDK4* | 88 | 1.89 (1.57 - 2.40) | 0.40 (0.30 - 0.54) | 0.21 (0.15 - 0.29) | **<0.0001** |
| *RELA* | 85 | 2.95 (2.57 - 3.18) | 2.88 (2.58 - 3.31) | 0.99 (0.89 - 1.15) | 0.86 |
| *TGFB2* | 77 | 0.07 (0.04 - 0.10) | 0.07 (0.05 - 0.11) | 1.14 (0.96 - 1.51) | **0.0015** |
| *TLR4* | 87 | 2.55 (1.94 - 3.38) | 2.50 (1.98 - 3.07) | 0.96 (0.79 - 1.19) | 0.26 |
| *TNF* | 88 | 0.98 (0.79 - 1.18) | 1.05 (0.90 - 1.31) | 1.11 (0.91 - 1.29) | **0.0107** |
| *TNFRSF1A* | 88 | 3.67 (3.09 - 4.56) | 3.51 (3.05 - 4.08) | 0.94 (0.76 - 1.20) | 0.12 |
| *TNFRSF1B* | 88 | 21.47 (17.14 - 28.11) | 18.67 (14.45 - 20.75) | 0.89 (0.64 - 1.09) | **0.0002** |
| **Lipids** |  |  |  |  |  |
| *ABCA1* | 77 | 0.09 (0.06 - 0.12) | 0.13 (0.10 - 0.17) | 1.36 (1.04 - 1.93) | **<0.0001** |
| *ABCG1* | 85 | 0.29 (0.23 - 0.38) | 0.38 (0.29 - 0.46) | 1.34 (0.87 - 1.64) | **0.0002** |
| *CD36* | 85 | 7.07 (6.22 - 8.85) | 7.79 (6.82 - 9.49) | 1.12 (0.96 - 1.33) | **0.0013** |
| *CPT1A* | 85 | 3.04 (2.26 - 3.64) | 2.24 (1.83 - 2.71) | 0.72 (0.56 - 1.00) | **<0.0001** |
| *CPT1B* | 84 | 0.10 (0.08 - 0.14) | 0.09 (0.08 - 0.13) | 0.90 (0.70 - 1.19) | 0.06 |
| *CRAT* | 86 | 1.84 (1.45 - 2.26) | 1.73 (1.32 - 2.27) | 0.97 (0.77 - 1.16) | 0.47 |
| *HMGCR* | 87 | 1.70 (1.50 - 2.06) | 1.71 (1.48 - 2.01) | 1.02 (0.85 - 1.23) | 0.77 |
| *LDLR* | 76 | 0.23 (0.19 - 0.28) | 0.20 (0.17 - 0.28) | 0.93 (0.77 - 1.26) | 0.47 |
| *LIPE* | ND | ND | ND | ND | ND |
| *NAMPT* | 85 | 1.22 (0.97 - 1.44) | 1.11 (0.92 - 1.36) | 0.90 (0.78 - 1.27) | 0.15 |
| *PLIN2* | 82 | 1.44 (1.23 - 1.73) | 1.06 (0.91 - 1.29) | 0.76 (0.61 - 0.94) | **<0.0001** |
| *PPARA* | 87 | 0.77 (0.64 - 0.89) | 0.79 (0.69 - 0.90) | 1.05 (0.87 - 1.21) | 0.47 |
| *PPARD* | 88 | 1.96 (1.76 - 2.27) | 1.94 (1.70 - 2.21) | 0.97 (0.82 - 1.16) | 0.61 |
| *SREBF1* | 82 | 1.15 (0.84 - 1.33) | 1.13 (0.91 - 1.69) | 1.18 (0.70 - 1.55) | 0.49 |
| *UCP2* | 86 | 43.74 (35.75 - 53.24) | 38.72 (33.15 - 44.22) | 0.90 (0.80 - 1.03) | **0.0016** |

^a^q<0.05 (FDR<5%) was considered significant.
